# Surface and Subsurface Quality of Titanium Grade 23 Machined by Electro Discharge Machining

**DOI:** 10.3390/ma15010164

**Published:** 2021-12-27

**Authors:** Panagiotis Karmiris-Obratański, Emmanouil L. Papazoglou, Beata Leszczyńska-Madej, Krzysztof Zagórski, Angelos P. Markopoulos

**Affiliations:** 1 Department of Manufacturing Systems, Faculty of Mechanical Engineering and Robotics, AGH University of Science and Technology, 30-059 Cracow, Poland; zagkrzys@agh.edu.pl; 2 Laboratory of Manufacturing Technology, School of Mechanical Engineering, National Technical University of Athens, 15780 Athens, Greece; mlpapazoglou@mail.ntua.gr (E.L.P.); amark@mail.ntua.gr (A.P.M.); 3 Department of Materials Science and Non-Ferrous Metals Engineering, Faculty of Non-Ferrous Metals, AGH University of Science and Technology, 30-059 Cracow, Poland; bleszcz@agh.edu.pl

**Keywords:** EDM, white layer, microhardness, EDX maps, microstructure’ Titanium Grade 23, Response Surface Method, ANOVA

## Abstract

Electrical Discharge Machining (EDM) is a non-traditional cutting technology that is extensively utilized in contemporary industry, particularly for machining difficult-to-cut materials. EDM may be used to create complicated forms and geometries with great dimensional precision. Titanium alloys are widely used in high-end applications owing to their unique intrinsic characteristics. Nonetheless, they have low machinability. The current paper includes an experimental examination of EDM’s Ti-6Al-4V ELI (Extra Low Interstitials through controlled interstitial element levels) process utilizing a graphite electrode. The pulse-on current (I_P_) and pulse-on time (T_on_) were used as control parameters, and machining performance was measured in terms of Material Removal Rate (MRR), Tool Material Removal Rate (TMRR), and Tool Wear Ratio (TWR). The Surface Roughness (SR) was estimated based on the mean roughness (SRa) and maximum peak to valley height (SRz), while, the EDMed surfaces were also examined using optical and SEM microscopy and cross-sections to determine the Average White Layer Thickness (AWLT). Finally, for the indices above, Analysis of Variance (ANOVA) was conducted, whilst semi-empirical correlations for the MRR and TMRR were given using the Response Surface Method (RSM). The results show that the pulse-on time is the most significant parameter of the machining process that may increase the MRR up to 354%. Pulse-on current and pulse-on time are shown to have an impact on the surface integrity of the finished product. Furthermore, statistics, SEM, and EDX images on material removal efficiency and tool wear rate are offered to support the core causes of surface and sub-surface damage. The average microhardness of the White Layer (WL) is 1786 HV.

## 1. Introduction

Electro Discharge Machining (EDM) is a technologically advanced, precision machining method. EDM is mainly used in industries and research fields of aerospace, medical, dental, and automotive engineering in order to manufacture ceramics [1], modify surface or the sub-surface microstructure [2,3] as well as optimize the machining parameters [4]. The process involves triggering a spark-generated erosion process between two electrodes, one of which is the tool and the other is the workpiece [5]. An electrostatic field is generated due to a maintained gap between the anode and the cathode (tool and the workpiece material respect), resulting in an emission of electrons. Thus, a discharge in the dielectric is breaking the electrons into positive ions and electrons with higher velocities. During this process, the material is removed using a series of thousands of electrical discharges between the electrodes. The generated temperature during the discharge phase could rise locally up to 12,000 K [6]. Many researchers study the machining parameters that involve the influence of pulse-on current (I_P_), pulse on (T_on_) and pulse-off time (T_off_), gap voltage, as well as the Material Removable Rate (MRR) and the Tool Wear Ratio (TWR) [7,8,9].

Die-sinking EDM as an unconventional and high-precision machining method has been successfully used to machine difficult-to-cut materials such as ceramics, cast alloys, superalloys, and among others, titanium alloys. Many industries currently use titanium and its alloys due to their desirable mechanical properties such as high specific strength, excellent corrosion resistance, and, more importantly, its low density compared to nickel and steel alloys [10]. Titanium alloys, on the other hand, have poor machinability owing to their low heat conductivity, strong chemical reactivity, and low elasticity modulus, making them difficult to cut. As a result, non-traditional machining methods such as EDM are often used in their machining [11,12]. More specifically, Wang et al. [13] investigated the influence of the dielectric during EDMed surface of titanium alloy. The results revealed that compound dielectric could achieve higher MRR and TWR comparing to kerosine [13] and lower Surface Roughness (SR) compared to distilled water. Hiui et al. [14] performed a comparative study with and without cryogenic cooling of the electrode during EDM of Titanium Grade 5. The experimental results show that a cryogenically treated electrode could improve the SR, decrease the TWR and the crack density [15,16]. Further experimental research on Titanium Grade 5 using EDM was carried out by Hasçalk and Caydas [17,18], in which they evaluated four different electrode materials: copper (normal and cryogenically treated), tungsten, and graphite (all of which were used in the same experiment). The machining performance was evaluated in terms of MRR, White Layer (WL) development, and its properties, and crack density, among other factors. Finally, it was determined that the cryogenically treated electrode outperformed the other electrodes in terms of enhanced MRR, improved surface polish, increased WL hardness, and associated lower Surface Crack Density (SCD). By using the Taguchi statistical method, researchers try to optimize the machining parameters of titanium alloys [19,20]. Results analysis shows that increasing pulse-on current is associated with increased pulse and heat energy, resulting in higher MRR and the SR. However, an increase of the pulse on-time has a great contribution on the TWR.

It is necessary to create a model between the process parameters and the response measure in order to better understand the behavior of the process. The Relative Wear Ratio (RWR) optimization model of Ti-685 during EDM operation was investigated by Agarwal et al. [21]. In order to increase the surface quality, a higher RWR value is required. They discovered that TWR dropped when pulse-on current rose but increased as pulse-on time and duty factor increased [22,23]. Bhaumik and Maity [24] performed, based on statistical Response Surface Method (RSM), a face-centered central composite experimental design to analyze the influence of the T_on_, I_P_, gap voltage, and Duty factor on the process performance of Titanium Grade 6 alloy machined by EDM. The authors used three different electrodes during the machining process. They reported that higher MRR could be achieved by using brass and zinc electrodes compared to commonly used copper electrodes, but the copper electrode produced comparatively better Surface Quality (SQ). In addition, for all the electrodes, the highest MRR was achieved at the 20A pulse-on current.

A couple of studies investigate the physical and micromechanical behavior of the surface and subsurface later synthesized by the Electrical Discharge Machining process. Holsten et al. [25] investigated the influence of polarity on the subsurface of titanium alloys machined by sink-EDM. The results pointed out that the anodic tool polarity produced a more uniform, hard, and brittle sublayer with a Face-Centered-Cubic (FCC) structure rich in carbon. In his study, Grigoriev et al. [2] machined X10CrNiTi18-10 steel by wire-EDM with water as a dielectric medium. The subsurface analysis revealed a nanomodification of the layers by combining elements between the wire electrode and the workpiece. Additionally, steel sublayers tend to engender integral stresses due to their low thermal conductivity combined with a high coefficient of linear explanation. Finally, Basak et al. [26] performed a micromechanical characterization of a grade 5 titanium superficial layer synthesized during the wire-EDM process. The brittle structure beneath the recast layer was examined using in-situ micro-pillars compression and nano-indentation techniques with SEM and TEM microscopy. The average thickness was around 10 µm with the presence of Cu and Zn elements transferred from the electrode among various carbides such as Ti24C15, TiC, and Al2Ti4C2. As a result of the rapid heating and quenching, the contentious Grain Boundary (GB) ribs of the second wetted phase were formed, resulting in the formation of a brittle structure. Additionally, the tensile stresses are formed parallel to the surface, resulting in micro-cracks due to titanium’s low thermal conductivity.

Within the relevant area of EDM titanium alloy machining, the purpose of the current article is to provide a thorough analysis of how the major machining parameters, i.e., the pulse-on current and the pulse-on time, influence the process. Ti-6Al-4V ELI was machined using EDM, with the graphite electrode being used as an electrode in a series of tests that were carried out. The acquired findings and their following statistical analysis offer valuable information that may be used not only for future research reasons but also in a more practical manner in the field of medicine. The machining performances were estimated in terms of MRR, Tool Material Removal Rate (TMRR), and TWR, while the machined surface roughness (SR) was measured in terms of mean roughness (SRa), maximum peak to valley height (SRz), in accordance with the ISO 25178-2 specifications. These two SR indicators were chosen as representative indexes of the machined surface roughness based on the relative literature, but also according to the physical meaning and practical value of SRa and SRz. Obviously, the SRa can be used as a basic indicator of the surface quality, and the need for post-process in order for the workpiece to meet probable roughness requirements. Moreover, the SRa constitutes a more macroscopic and general SR index since conceptually it pertains to the mean roughness, and hence, this macroscopic approach is very helpful in EDM, which is by definition a chaotic and non-deterministic process in micro-scale. At the same time, the SRz, which stands for the maximum peak to valley height, is a very useful index to assess and estimate the highest peaks and valleys distance. For a process like EDM where the obtained surfaces are not entirely uniform, the definition of SRz is very useful since topical material accumulation/deposition, and/or the formation of a significant deep crater can be estimated and measured. Furthermore, the subsurface was examined using an optical microscope to determine the Average White Layer Thickness (AWLT), while SEM microscopy and Energy Dispersive X-Ray analysis (EDX) were used to identify the elemental composition of materials. Additionally, by using Vickers micro-hardness technique, the hardness of the WL was defined. Finally, an Analysis of Variance (ANOVA) was conducted for each of the indices mentioned above and using the Response Surface Method (RSM), semi-empirical correlations between the machining parameters and the MRR and TMRR were suggested.

## 2. Materials and Methods

Many titanium-based compounds have been created in recent decades for a variety of uses. Ti-6Al-4V ELI (Grade 23) is the most commonly utilized high-strength titanium alloy at the moment. It was developed in the 1950s in the USA and since then widely used in the automotive, marine, chemical, and aerospace sectors and medicine. Since it is an α + β phase alloy, a beta fraction volume ranges from 5 to 40% at room temperature. If the percentage of β-stabilizing elements is raised further to the point where β no longer changes to martensite following rapid quenching, the alloys remain in the two-phase field and are classified as metastable beta alloys. It should be noted that these alloys may nevertheless exhibit an equilibrium alpha volume fraction of more than 50%. Finally, single-phase betta alloys represent the end of the titanium alloy alloying scale. For that reason, one of the most significant problems during the production of these alloys is the control and optimization of the morphology of the alpha phase to gain a high effective microstructure. The following Table 1 and Table 2 contain the mechanical and chemical composition of the utilized titanium and graphite material.

**Figure 1:**
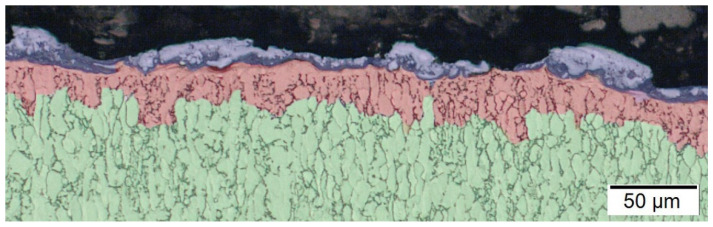
Microstructure evaluation of Titanium Grade 23 machined by EDM.

Under equilibrium circumstances, the chemical compositions of the α and β phases vary as the temperature decreases in the two-phase field. At lower temperatures, vanadium significantly enriches and therefore stabilizes the β phase. A slowly cooled specimen can reveal a tiny seam surrounding the coarse and light-colored lamellae in a metallographic figure, as can be seen with the green color in the following Figure 1. It can be seen that the microstructure of the base material is coarse, which is more resistant to creep and fatigue crack growth. During the machining process, the high cooling rates from temperatures above the martensitic start (MS) temperature and transformation into martensite through the two-phase field (HAZ) can be seen. As shown in Figure 1 with the red color, the martensitic initiation temperature varies based on the initial microstructure homogeneity. One of the most significant problems in the usage of this alloy is the control and optimization of the morphology of the alpha-phase. Thermomechanical processing is a highly effective technique of enhancing the microstructure, such as regulating the size and aspect ratio of the lamellar phase, optimizing the phase and chemistry of the primary and secondary phases, and controlling the phase morphology. Hot deformation of the Ti-6Al-4V alloy in the phase-field leads to significantly large prior-grains, phase development during cooling, and martensite production upon fast quenching. Because the primary phase restricts the development of phase, processing in the α + β phase-field yields a considerably finer α + β structure. While processing in the β phase field, microstructural development may entail mechanical deformation of the equiaxed β grains, possibly dynamic or meta-dynamic recrystallization during processing, and the phase β to α change following deformation when cooling.

**Figure 2:**
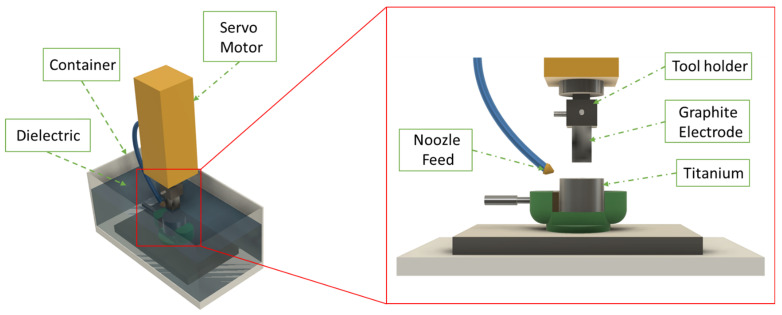
Graphical representation of EDM setup.

As a workpiece, a 47 mm Ti-6AL-4V ELI (Grade 23) ride was utilized in the present experimental research, which was cut into slices of 10 mm thickness. Titanium Grade 23 (also known as alpha-plus-beta phase titanium) is extensively utilized in the aerospace and biomedical sectors. The tests were carried out with the help of a rectangular graphite electrode with nominal dimensions of 38 mm^2^ by 38 mm^2^ cross-section area and 200 mm length. All of the tests were carried out using a Swiss-made Roboform Agie Charmilles 350Sp EDM (GF Machining Solutions, Biel, Switzerland; the experimental setup is graphically shown in Figure 2.

Full-scale experiments were carried out using the pulse-on current and pulse-on time as control parameters since, according to the literature [27,28,29,30,31], these machining parameters are the ones that have the most significant influence on process performance [28,29,30,31]. A constant Duty Factor of 0.5 was maintained throughout the experiment, and square pulses with open and close circuit voltages of 120 and 30 V, respectively, were used to generate the signals. It was decided to use hydrocarbon oil as the dielectric fluid, which was appropriately routed into the working tank under continuous pressure in order to ensure effective debris flushing. The nominal cutting depth was chosen at 0.5 mm in order to allow for the development of the machined surface features to their maximum potential. Finally, during the intervals between tests, the graphite electrode was dried out, allowing the real electrode wear to be evaluated after the dielectric fluid had been removed from the electrode. The experimental parameters are given in full in Table 3, which may be found here. The MRR, TMRR, and TWR were computed in accordance with Equations (1)–(3), which were written as follows:

(1) $$\mathrm{MRR}=\frac{W_{\mathrm{st}}-W_{\mathrm{fin}}}{t_{\mathrm{mach}}}\cdot \frac{1}{\rho_{w}}$$

(2) $$\mathrm{TMRR}=\frac{{\mathrm{El}}_{\mathrm{st}}-{\mathrm{El}}_{\mathrm{fin}}}{t_{\mathrm{mach}}}\cdot \frac{1}{\rho_{\mathrm{el}}}$$

(3) $$\mathrm{TWR}=\frac{{\mathrm{El}}_{\mathrm{st}}-{\mathrm{El}}_{\mathrm{fin}}}{W_{\mathrm{st}}-W_{\mathrm{fin}}}$$

with MRR in mm^3^/min, TMRR in mm^3^/min, TWR in gr/gr, ρ_w_ and ρ_el_ the workpiece and electrode material density respectively in gr/mm^3^, t_mach_ is the machining time in min, W_st_ and W_fin_ are the workpiece weights before and after the machining in gr, while El_st_ and El_fin_ are the electrode’s weights before and after the machining respectively in gr.

A TOPO 01P (IOS, Cracow, Poland) contact profilometer equipped with an induction measuring head with a cone-shaped diamond tip of 2 μm radius and a 90° apex angle was used to measure SR. The device has a confocal sensor with a range of 130 μm and a vertical resolution of 8 nm. Cut-off lengths are established using a Gaussian filter based on Fourier transformation, which means that λs filtering is used to reduce microroughness caused by instrument or environmental noise, and λc filter is used to separate the waviness from roughness, and thus roughness can be defined. With respect to the relevant norms, SR measurements were taken on randomly selected machined surfaces, and a 2.5 λs filter was used with a cutting length of 8.0 mm. The 3D surface map was created by scanning an area of 1.25 mm by 10 mm at a speed of 0.5 mm/s over 101 consecutive routes. The resulting data were examined in accordance with ISO 25178-2. The SR measurements were cross evaluated using a VHX-7000 ultra-deep-field microscope (KEYENCE, Mechelen, Belgium) and a 20–2000 objective using Focus Variation Microscopy (FVM).

Keeping in mind that for certain machining parameters the deviation in the obtained results is significant, along with the Boxplots and the mean values estimation, the test of statistically significant difference is required. To estimate the statistically significant difference between factors’ level means there are a number of methods. In the current study the Fisher’s least significant difference (LSD) method for multiple comparisons was employed. That way, the real effect and impact of each individual parameter on the machining can be more accurately and reliably defined, avoiding any misleading estimations based only on the study of the Boxplot diagrams. Finally, the statistical analysis is completed by utilizing the RSM to define and propose semi-empirical correlations for the MRR and TMRR with the machining conditions. In RSM, the best fit model may include linear, squared, and cross-product terms of the independent variables, with the respective coefficients estimated based on the least square method. It is widely accepted, and aiming in the least complex equations—relations, in the RSM only the most significant terms are to be considered. Nevertheless, there is no general rule regarding the optimum order of the model, and hence, the trial-and-error method is utilized to define the necessary and most suitable terms that have to be included. The RSM can be mathematically described by the following formula:

(4) $$f\left(x_{1},x_{2},\ldots x_{k}\right)=a_{o}+\underset{\mathrm{linear}\;\mathrm{terms}}{\underbrace{\overset{k}{\underset{i=1}{\sum }}a_{i}x_{i}}}+\underset{\mathrm{squared}\;\mathrm{terms}}{\underbrace{\overset{k}{\underset{i=1}{\sum }}a_{\mathrm{ii}}x_{i}^{2}}}+\underset{\mathrm{cross}-\mathrm{product}\;\mathrm{terms}}{\underbrace{\underset{i<j}{\sum \sum a_{\mathrm{ij}}x_{i}x_{j}}}}$$

## 3. Results and Discussion

The following Table 4 contains the experimental results. A detailed analysis on the results of each parameter will contain a Fisher test as well.

### 3.1. Material Removal Rate, Tool Removal Rate and Tool Wear Ratio

It is well known and proven that the MRR and the TMRR are strongly affected by the machining power and the per pulse energy (i.e., the pulse-on current and the pulse-on time). Nevertheless, and keeping always in mind the non-linear behavior of EDM, the intuitive assumption that higher power or energy will compulsorily lead to higher MRR and TMRR is wrong and may become misleading. Indeed, higher machining power and/or per pulse energy may lead to increased molten material volume and hence increased MRR, however, there are also three underlying main physical mechanisms that are taking place simultaneously which strongly affect and limit the process, namely, the plasma channel growth over time, the debris concentration, and carbon decomposition. In more detail, the plasma channel that is formed between the electrode and the workpiece is expanding over time, consuming significant amount of the discharged energy, while, at the same time, and as the plasma channel is expanded, its increased radius results in lower densities of energy and power [5,32,33]. The second parameter that strongly affects the MRR and the TMRR is the debris concentration in between the electrode and the workpiece. These debris, from one point and onwards, cannot be efficiently flushed away, its concentration is increased and negatively affects the efficiency of machining process. Apart from the fact that an amount of energy is consumed and spent in their remelting, destabilization of the process may be also resulted along with arcing conditions [34]. Finally, the decomposition of the dielectric fluid and the bond of the carbon on the electrode and workpiece surfaces strongly affect the MRR and the TMRR. The carbon deposition on the electrode surface may be beneficial for the TMRR as it acts like a “protective layer”, nevertheless, it may also not be beneficial for the MRR resulting in its decrease [21,35]. From the previous brief theoretical analysis, it is deduced that the EDM has a “slight peculiar” and nonlinear behavior [21,25,35], and thus, the effect of the machining parameters (i.e., pulse-on current and pulse-on time) have to be studied carefully and in depth, avoiding superficial and generalizing approaches.

**Figure 3:**
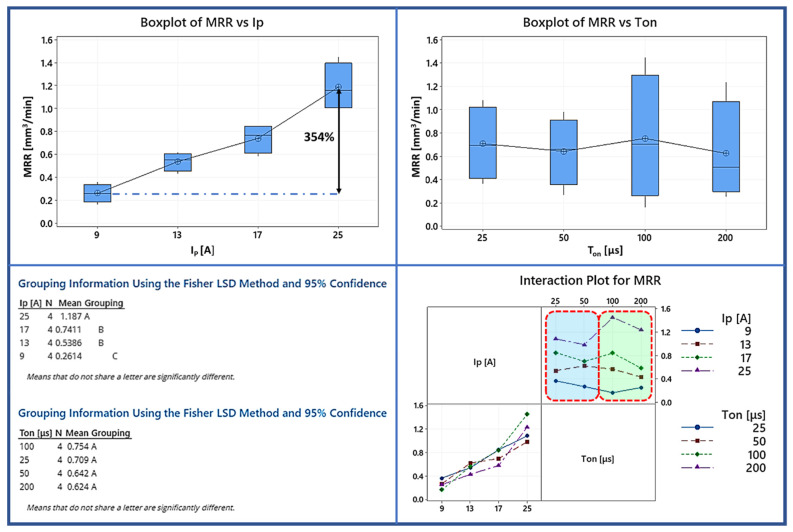
Box plot, interaction plot of MRR and the results of the Fisher statistically significant test.

In Figure 3 the Box plot and the interaction plot of MRR are depicted along with the results of the Fisher test for the statistical significance between the different machining parameters. From its data and graphics (Figure 3), it is deduced that the pulse-on current mainly and strongly affects the MRR, while, the pulse-on time does not have any statistically significant impact on it. More specifically, as the I_P_ increased from 9 to 25 A the mean MRR increased by approximately 354%. The value ranges that have significant in between differences are 9, 13–17, and 25 A (i.e., between 13 and 17 did not recorded a statistically significant difference). One final interesting point concerning the effect of I_P_ on MRR is that for all the pulse-on times (T_on_) the MRR increased as function of I_P_ in almost linear ways (see interaction plot). Nevertheless, it has to be pointed out that this behavior corresponds only to the specific range of machining conditions, and it would be a mistake to arbitrarily generalize those conclusions. Recording the pulse-on time, it is proved that it does not affect the mean MRR in a statistically significant way. At the same time, and by studying the interaction plot, some interesting conclusions can be deduced. The aforementioned peculiar non-linear behavior is confirmed as we find that in some cases, higher pulse-on time (i.e., higher per pulse energy) results in lower MRR (e.g., for 25 A between 100 and 200 μs). This behavior should not surprise us, since it has been recorded in literature in machining with EDM and especially in machining titanium alloys [36,37]. One more interesting remark is the deviation of MRR values for different pulse-on times. For lower pulse-on times (25 and 50 μs)-blue area) MRR values less deviate depending on the pulse-on current, while, for higher pulse-on times (100 and 200 μs-green area) this deviation is increased, implying that the combination of the machining parameters becomes more important and affects more the machining outcome for higher T_on_ and per pulse energies. This behavior, i.e., that the MRR is increased for higher currents, while higher pulse-on times result in a decrease in MRR, can be interpreted by focusing on the fundamental material removal mechanism. The increased machining power through higher pulse-on current consequences more intense topical conditions of pressure, with the removal of the melted material being favored and enhanced that way. Thus, the process becomes more efficient and the MRR is increased, not only because of the higher machining power that results in a greater amount of material volume to be melted, but also because the material removal process is becoming more efficient. On the other hand, and keeping in mind that the Duty Factor remains constant, the pulse-on time does not affect the machining power or the total energy (for a given pulse-on current), but only their temporal and spatial distribution. Higher pulse-on time means that each pulse lasts more, and thus more energy is delivered to a single spot. Nevertheless, although this accumulated energy results in the melt of more material, this melted material cannot efficiently be flushed away, but it is resolidified, forming a thicker WL (see Section 3.3). Hence, although the same total amount of energy is consumed and the per-spark energy is increased for higher pulse-on times, the MRR is not increased (even decreased), since the material removal becomes less efficient, while portion of the energy may be consumed for the remelting of the resolidified material and the expansion of the plasma channel over time. As an overall conclusion, and as a basic rule of thumb, it can be said that in order to increase the MRR in machining of titanium alloys the most suitable way is by increasing the pulse-on current and not the pulse-on time. Nevertheless, other parameters, like the TMRR and the obtained surface quality and roughness have always to be considered in machining planning.

**Figure 4:**
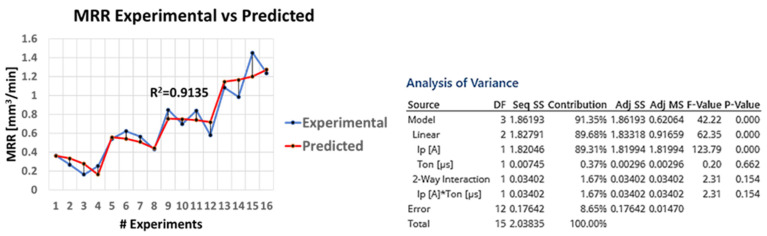
Plot of experimental MRR vs predicted MRR along with model’s ANOVA.

It is also of extreme interest and with practical value as well, the capability to predict the MRR according to the machining parameters. Based on the RSM and by trial-and-error method, the three most important parameters were finally employed (i.e., the I_P_, T_on_, and I_P_·T_on_—see Figure 4), and the following semi-empirical relation is proposed to correlate the MRR with the I_P_ and the T_on_:

(5) $$\mathrm{MRR}=-0.025+0.04611I_{P}-0.00218T_{\mathrm{on}}+0.000116I_{P}T_{\mathrm{on}}$$

with MRR in mm^3^/min I_P_ in A and T_on_ in μs.

At first, a high fit between the model and the experimental results can be achieved with R^2^ over 0.91, implying that semi-empirical relations can be utilized to sufficiently predict the MRR during the machining planning. Moreover, the significance of the pulse-on current for the MRR in comparison with the pulse-on time, is confirmed since the contribution of the I_P_ term is almost 90%. Finally, the vague and in some cases not beneficial effect of T_on_ on MRR is depicted on the extremely low and negative value of the T_on_ coefficient (i.e., −0.00218), when the respective coefficient of I_P_ is positive, and an order of magnitude higher (i.e., 0.04611).

**Figure 5:**
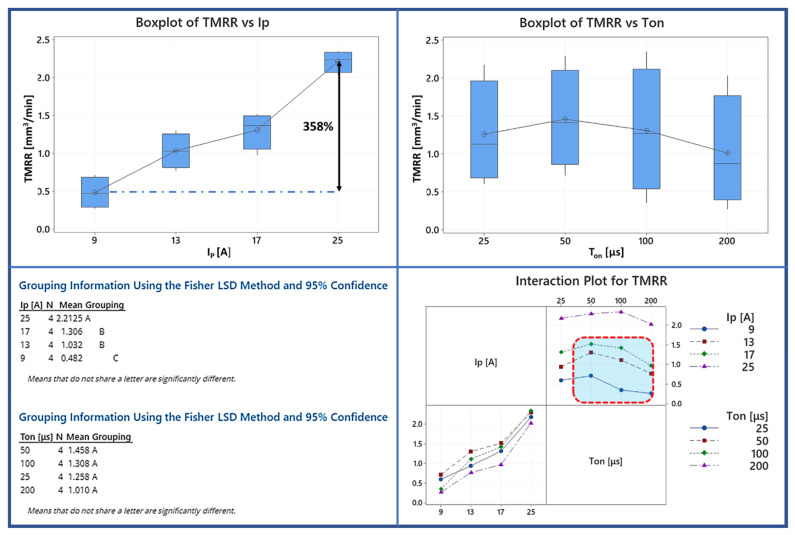
Box plot, interaction plot of TMRR and the results of the Fisher statistically significant test.

In Figure 5 the Box plot and the interaction plot of TMRR are depicted along with the results of the Fisher test for the statistical significance between the different machining parameters. Based on the results for TMRR, it is easily concluded that the graphite electrode behaves similarly to the workpiece in term of the volumetric material removal rate. Between 9 and 25 A an increase of 358% in TMRR was measured (almost the same with the corresponding of MRR), while, for all the pulse-on times, the increase of TMRR in respect of the pulse-on current is almost linear again. Moreover, like in MRR, there is not a statistically significant difference in the mean TMRR between 13 and 17 A, while, between the rest of the I_P_ values a statistically significant difference emerged. Regarding the impact of the pulse-on time on TMRR, again it is ambiguous. At first, there is no statistically significant difference in mean TMRR for the different pulse-on times (T_on_), which can be reasonably attributed to the high level of deviation depending on the combination of I_P_ and T_on_. Finally, based on the interaction plot, we find that the TMRR, for each I_P_ reaches a peak value and then, for higher pulse-on times it is constantly decreased. For 9, 13, and 17 A the peak TMRR was measured for 50 μs Ton, while for 25 A, this peak was shifted at the 100 μs. Again, and as was discussed earlier regarding the decrease of MRR for higher pulse-on times (T_on_), the main cause for the lower TMRR for higher pulse-on times is the increased consumption of energy from the plasma channel as it is expanded over time. In the case of the graphite electrode, the main material removal mechanism from the electrode is the ablation, thus, it is reasonable and justified to be said that the flushing efficiency does not significantly affect the TMRR. Hence, the decrease of TMRR can be mainly attributed to the less portion of energy that is absorbed by the electrode, since the plasma channel, and the “plasma bubble” that is formed consume for higher pulse-on times (T_on_) increased amount of energy. The important conclusion that is deduced, and mainly because of the high deviation in the TMRR values for the same pulse-on time, is that, during the machining planning, the combination of the I_P_ and T_on_ has to be carefully chosen and decided, in order for the minimum TMRR to be achieved. Lower TMRR does not only result in higher efficiency, but also higher dimensional accuracy and precision since it corresponds to the material volume removal from the electrode, and hence, the difference in electrode’s nominal dimensions during the machining.

Again, it would be very practical, an estimation of the TMRR can be done depending on the machining parameters. Following the same already mentioned methodology (RSM and trial and error method to define the most important terms) a semi-empirical model to correlate the TMRR with the pulse-on current and pulse-on time emerged:

(6) $$\mathrm{TMRR}=-0.443+0.10533I_{P}+00350T_{\mathrm{on}}-0.000023T_{\mathrm{on}}^{2}$$

with TMRR in mm^3^/min, I_P_ in A and T_on_ in μs.

**Figure 6:**
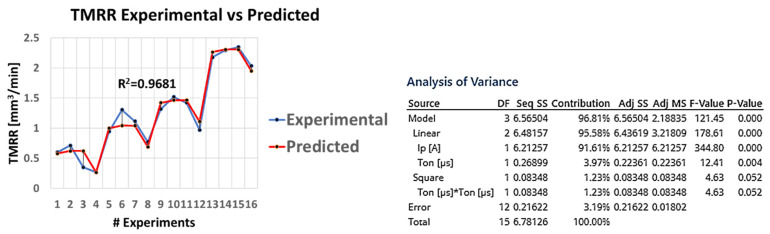
Plot of experimental TMRR vs predicted MRR along with model’s ANOVA.

The high value or the R^2^ (over 0.96) confirms the sufficient level of fit for the proposed semi-empirical model (Figure 6). The most important terms are again the I_P_ and the T_on_, but for the TMRR, the third most important term is now the T_on_^2^, while for MRR was the I_P_·T_on_. The significance of the pulse-on current is depicted on the positive, and an order of magnitude higher coefficient of I_P_ in comparison with these of T_on_ and T_on_^2^. Finally, the decrease of TMRR after a given pulse-on time value, is expressed through the positive coefficient for T_on_ and the negative for T_on_^2^.

**Figure 7:**
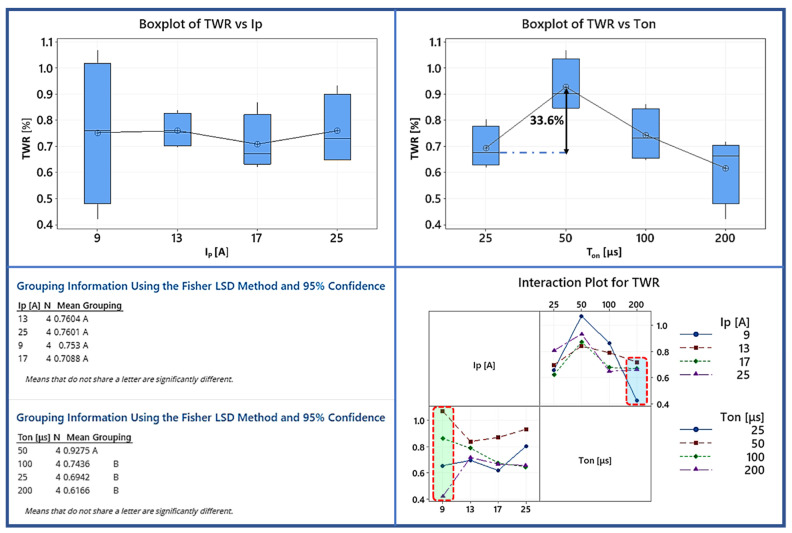
Box plot, interaction plot of TWR and the results of the Fisher statistically significant test.

The final machining performance index concerns the TWR. In practice, the TWR can be indirectly calculated based on the MRR and TMRR, nevertheless, it is quoted as a separate performance index for two main reasons. Firstly, for sake of completeness and in order for the results and conclusion to become more direct to the reader. Additionally, TWR is straight related with the economic feasibility of the EDM process as well as with its grade. The present electrode is an ultrafine graphite grade with average particle size of less than 10 microns providing high strength and resistance to damage. The high volumetric tool wear is associated with the development of high temperatures as well as with the grain size. MRR and TMRR are indexes of material removal in volumetric terms, while the TWR is the ratio of the material weight that is removed from the electrode and the workpiece. Hence, and taking in mind that materials are usually costed per mass, the TWR consists of a more representative and also significant index. Based on the Box plot of TWR in Figure 7 an interesting conclusion is deduced, namely that the pulse-on current does not have any significant impact on the mean TWR, while the pulse-on time seems to affect it more. Previously, the similar behavior of the electrode and the workpiece in respect of changes in I_P_ were presented, and now it is confirmed that the material removal by the electrode and workpiece changes in the same way not only in a qualitatively way, but also quantitively. Hence, there is no statistical difference in the mean TWR for different pulse-on currents. On the other hand, by studying the interaction plot, it has to be pointed out that for 9 A the deviation in TWR for different pulse-on times is significant, implying the sensitivity of the process’ efficiency for I_P_ 9 A. The TWR for 200 μs is 0.42 while for 50 μs it is 1.07, 154% higher, indicating that an efficient machining can be achieved by choosing specific and proper parameter combinations. The pulse-on time has a statistically significant impact on the mean TWR between 25 μs and the rest of the pulse-on times. The mean TWR increases 33.6% between 25 and 50 μs, while, based on the interaction plot, for 200 μs the deviation in TWR is again considerable for different pulse-on currents, rising the need for picking the optimum machining parameters combination.

From the above analysis of MRR, TMRR, and TWR, as a brief and summarized conclusion, it can be said that the parameter of the main and major impact is the pulse-on current, nevertheless, the pulse-on time has always to be taken into consideration during the machining planning since it can significantly affect the machining efficiency.

### 3.2. Surface and Subsurface Quality

#### 3.2.1. Surface Texture

This section will provide a detailed analysis of the SR and Average White Layer formation; Box plots, interaction plots, and Fisher test will help understand the complexity of this machining process. Nowadays, new materials with improved mechanical properties are constantly being developed. Rapid changes in temperature, with very high variation produce material melting and evaporation, resulting in craters on the material’s surface. A unique state of the surface’s geometric structure is established, with stereometry formed by superposition of traces of single electric discharges. Individual crater form and depth are primarily determined by the type of electric impulses (applied processing parameters), such as pulse-on current strength, pulse-on duration, pause time, and discharge voltage. The quantity of material degraded during a single discharge rises as pulse-on current intensity increases. The pulse-on time controls the quantity of heat energy given to the workpiece material as well as the amount of eroded material.

As previously stated, the workpiece removes a little bit of material with each spark, leaving behind a tiny crater. The entire amount of material removed is the consequence of hundreds of millions of sequential sparks. The acquired SR seems to be related to the morphological properties of the generated craters, which, according to the literature, are dependent on the machining parameters. In general, pulse energy has an effect on crater volume, with the pulse-on current mainly influencing crater depth and the pulse-on duration influencing crater breadth [5]. However, because of the fluctuating nature of EDM, the superimposition of subsequent craters, and the creation of the White Layer, the final SR is significantly more intricate. EDM is a chaotic phenomenon at the micro-scale (both spatial and temporal). Therefore, any effort at a purely deterministic description would be inadequate [37]. This discovery implies that the sparks are created randomly to some degree, and so the SR does not emerge from ordered craters but rather from randomly overlapping ones. Finally, just a fraction of the material that melts on each spark is removed, with the remainder re-solidifying on a workpiece surface. Simultaneously, ablated material that remains close to the surface may be re-condensed and attached to it, resulting in “debris adheres” across the surface. The re-solidified and re-condensed material forms an amorphous layer, often referred to as a White Layer, with more unique qualities than the mother material. The WL attributes (thickness and morphological features) are mostly determined by the machining settings (e.g., pulse-on current and duration), electrode and workpiece material, and dielectric fluid used.

**Figure 8:**
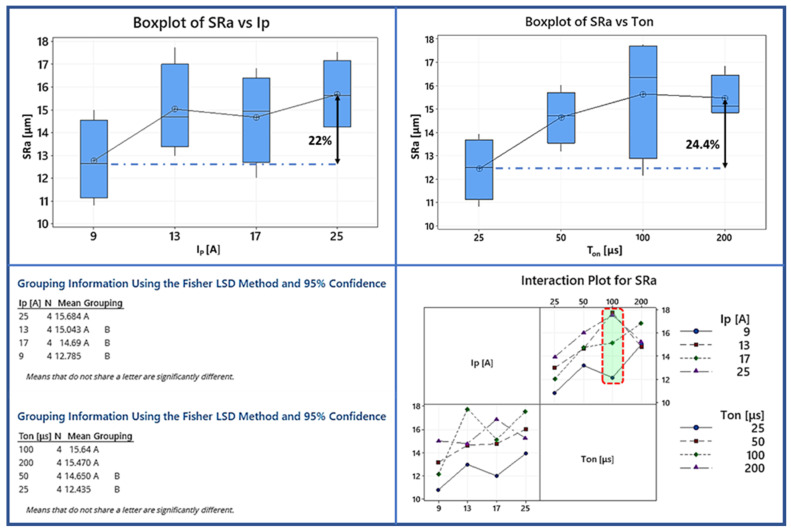
Box plots, Fisher test and interaction plot for the SRa.

The following Figure 8 shows the arithmetic mean of the roughness profile (SRa). SR is very critical in machining and especially in high precision machining. As it has been stated before, during EDM, complex and chaotic phenomena occur; hence, the SR is an outcome of the machining parameters. As the box plots show in Figure 8, the pulse-on time and the pulse-on current are the two most significant parameters affecting the SRa [8]. Both the box plot diagrams along with the interaction plot show a strong correlation with the increasing machining time and pulse-on current. As the pulse-on time increases from 9 to 25 A, the average increase of the SRa parameter is 24%. Although the box plot reveals that the increase of the Ra with regard to the T_on_ is almost linear, the interaction plot shows that at 100 µs, the combinations of 9 and 17 A decline the SRa growth rate. On the other hand, the combinations of 13 and 25 A at the same pulse-on time escalates and at then at the 200 µs with SRa values between 14.5 to 16 µm. When Figure 8 is examined to determinate the influence of pulse-on current on the SRa, it can be observed that as the I_P_ increases the SRa value increases up to 22%. The surface roughness is better in the lower pulse-on current as a result of shallower craters [17].

**Figure 9:**
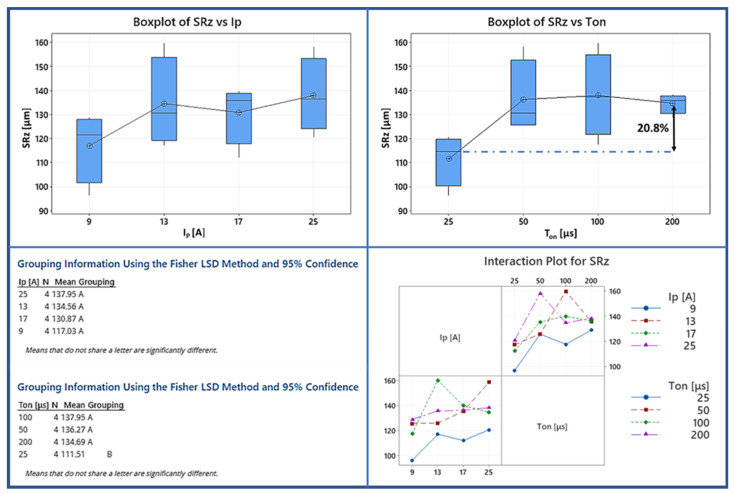
Box plot, interaction plot of TWR, and the results of the Fisher statistically significant test for SRz.

**Figure 10:**
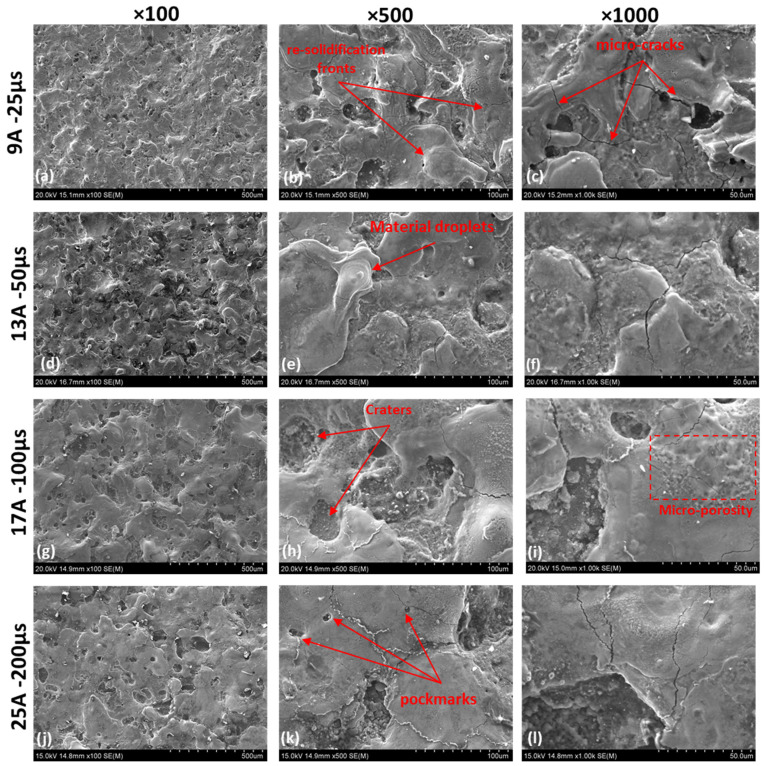
SEM images illustrating the workpiece surface in different magnification and machining parameters. For (**a**–**c**) 9 A—25 µs, (**d**–**f**) 13 A—50 µs, (**g**–**i**) 17 A—100 µs, and (**j**–**l**) 25 A—200 µs machining conditions.

The following Figure 9 illustrates the influence of machining parameters on the maximum height parameter (SRz) to have the overall picture of the surface texture. As it can be seen, by increasing the pulse-on time, the average SRz increases from 111 µm to 135 µm. It can also be observed on the SEM images in Figure 10 that as the pulse-on time and current increase, the cavities are more extensive but not necessarily deeper. There are three comments that could be made by analyzing the graphs regarding the SRz; Initially, there is no clear correlation between the pulse-on time and current. For example, the interaction plot shows that the combination of machining parameters of 13 A and 100 µs leads to higher SRz values compared with all the other machining parameters. Additionally, the Fisher test shows that the average SRz is almost constant to any change of the pulse-on current. Hence, it varies from 117.03 µm for the I_P_ of 9 A to 137 µm for the 25 A pulse-on currents. Finally, an observation worth mentioning regarding the variation of SRa and SRz depending on the machining parameters, is that the mean values of SRa and SRz have a statistically significant difference only between the extreme values of I_P_ and T_on_ (i.e., the mean SRa has statistically significant difference between 9 and 25 A pulse-on current and 25 and 200 μs pulse-on time, while the mean SRz is only between 25 and 200 μs). Hence it is deduced that although the mean SR values can be utilized as guidelines in machining planning, the specific SR emerged for each particular machining parameter combination. The difficulty in foreknowledge and accurately determining the SR is due to the complexity of the process, since the SR is the superposition of multiple craters, the material’s re-deposition and spattering, and the flow of the molten material that has not been efficiently removed on the workpiece surface.

#### 3.2.2. Surface Quality

The following Figure 10 shows SEM mix images from the machined surface. In general, the surfaces contain countless microcracks and micro-pores as a result of high temperature gradients and entrapped gases that occur during the rapid quenching EDM circles. This high temperature gradient manifests the residual stresses; when the stresses exceed the material’s tensile stress, the cracks are created [18,38]. Pulse-on current and time _(Ton)_ are associated with the surface crack density [39]. It is well known that titanium in general, has weak thermal conductivity and that the crack density is related to the material’s thermal properties [28].

The plasma channel’s heat vaporizes the dielectric liquid and a tiny fraction of the material during each circle, resulting in gas bubbles. When the current pulse is interrupted, and the plasma channel is extinguished, the gas bubble implodes on the discharge location. This implosion reduces the molten metal pool from the location even more, while a few gas bubbles contaminate the remaining molten pool throughout the solidification stage. Hence, WL contains both micro-porosity and pockmarks (see Figure 10i,k).

### 3.3. Subsurface Quality

#### 3.3.1. White Layer

**Figure 11:**
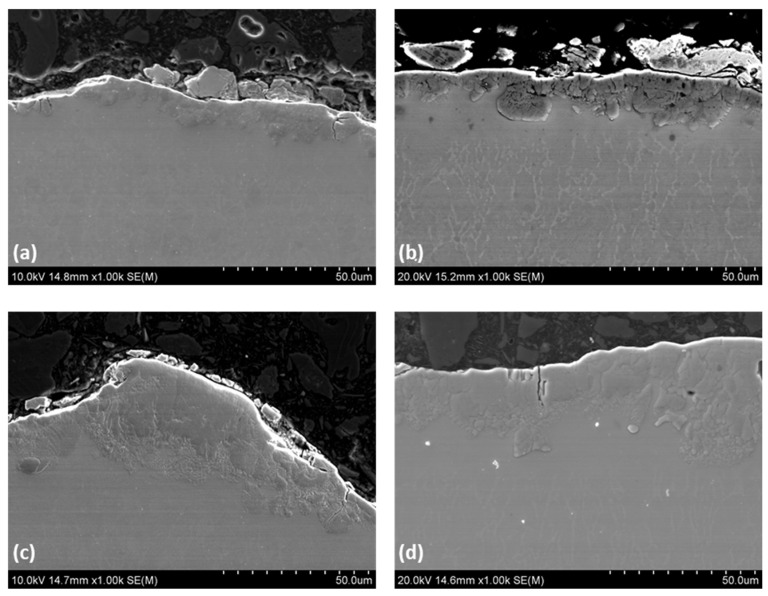
Cross section SEM images for (**a**) 9 A—25 µs, (**b**) 13 A—50 µs, (**c**) 17 A—100 µs, and (**d**) 25 A—200 µs machining conditions.

**Figure 12:**
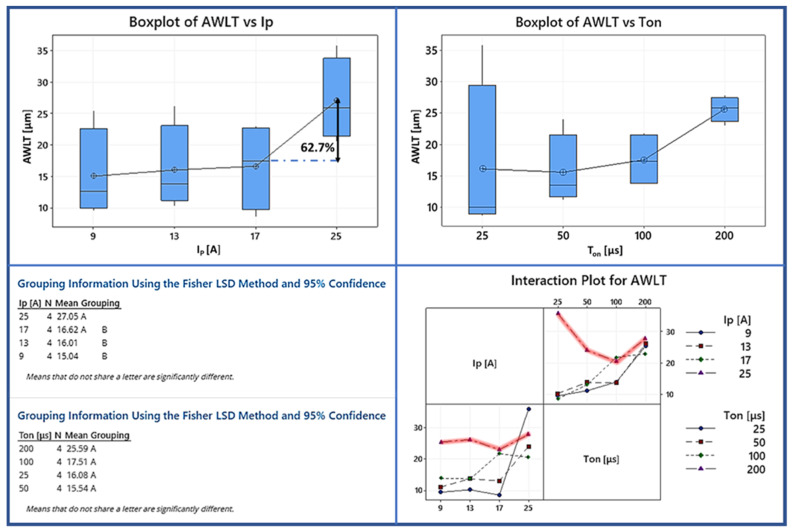
Analysis of the AWLT by using Fisher test, Box, and interaction plots.

As stated above, EDM is a suitable solution for machining difficult-to-cut materials for demanding applications; it is crucial to define the WL’s mechanical and chemical behavior. Most of the recent studies focused on the surface quality without considering the material’s changes during this process. Figure 10 shows the progressive change in WL features observed and qualitatively analyzed when increasing machining power and per-pulse energy are used. The WL is rather thin and uniform (Figure 11a) for pulse-on current and duration of 9 A and 25 μs, respectively. According to the boxplot (Figure 12), the average WLT remains stable at 16 μm for all pulse-on current combinations except I_P_ 25 A, which increases by 62 percent from 16 μm to 27 μm. For more intensive machining settings, as Figure 11c shows (i.e., 17 A and 100 μs), the WL recovered its thickness but remained its inhomogeneity, resulting in regions with significant WL thickness differences (areas with extremely thin WL and areas with thicker WL). Finally, at 25 A and 200 μs, at the most intense machining conditions in the present research, the white layer is becoming thicker and more irregular. By analyzing the interaction plot it can be seen that the machining conditions of the 25 A are in contrast with all the others. Particularly, the WL higher for the short pulse-on times. The only reasonable explanation for this phenomenon is, that the short pulses do not penetrate the material deep making the flushing efficiency difficult [29,33].

#### 3.3.2. Chemical Elements Distribution of the WL

**Figure 13:**
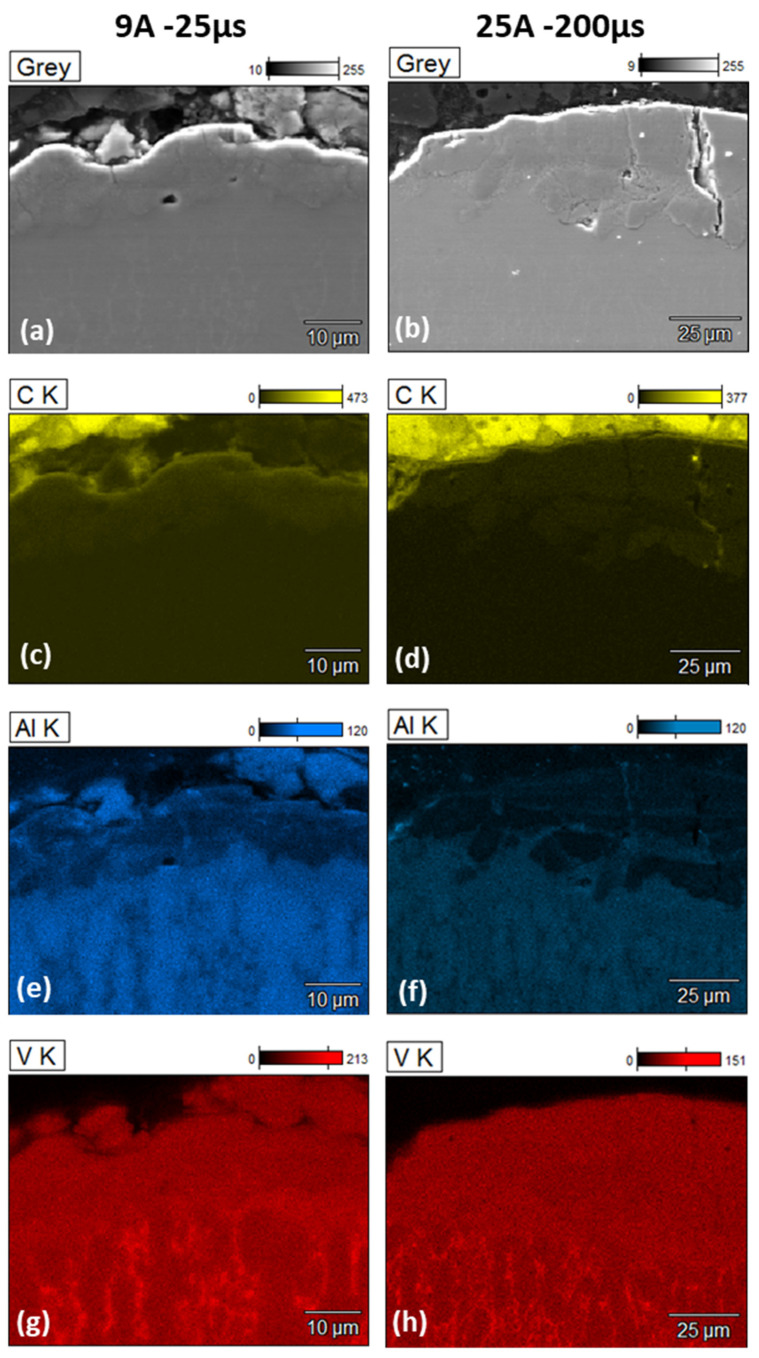
EDX chemical composition maps for 2 different machining conditions. For (**a**,**c**,**e**,**g**) 9 A—25 µs, and (**b**,**d**,**f**,**h**) 25 A—200 µs machining conditions. Yellow signals indicate the localization of carbon (**c**,**d**), blue signals indicate the localization of aluminum element (**e**,**f**), and red signals indicate the localization of vanadium element (**g**,**h**).

It is vital to determine the white layer’s metallurgical structure and chemical composition to comprehend its mechanical properties. In the initial stage, the chemical composition was studied. The distribution of elements in the white layer allows for the definition of homogeneity and the degree of contamination with elements from the parent material and wire electrode. In an EDX-map, Figure 13 depicts the typical element distribution of a surface machined with nine trim cuts. The EDX-map depicts the counts of a certain element in relation to its location in the rim zone. The more counts of an element at one point, the brighter the associated spot on the map. It is not feasible to quantify the whole chemical composition of the rim zone. In order to overcome these constraints, four typical EDX-analyses of the white layer for each condition were performed (see Figure 13). The average composition is shown in Table 2. The present material contains titanium as a base material, aluminum, vanadium, and graphite from the electrode. It is very interesting to compare the presented pictures c and d in Figure 13. There is a diffusion process from the electrode material to the WL. Particularly in lower energies, the diffusion process is higher, but there is a concentration of the transferred material into the cracks in higher energies. This situation could also be associated with the carbon that comes from the decomposition of the hydrocarbon dielectric [30].

Figure 13, pictures e and f show the presence of aluminum in the WL. As the energy per-pulse increases, the aluminum presence decreases. This occurrence is associated with the thermophysical behavior of the elements. Aluminum requires almost one-sixth of thermal energy for melting [40], where vanadium is considered more stable in the same range of melting temperatures [41,42].

### 3.4. Microhardness

**Figure 14:**
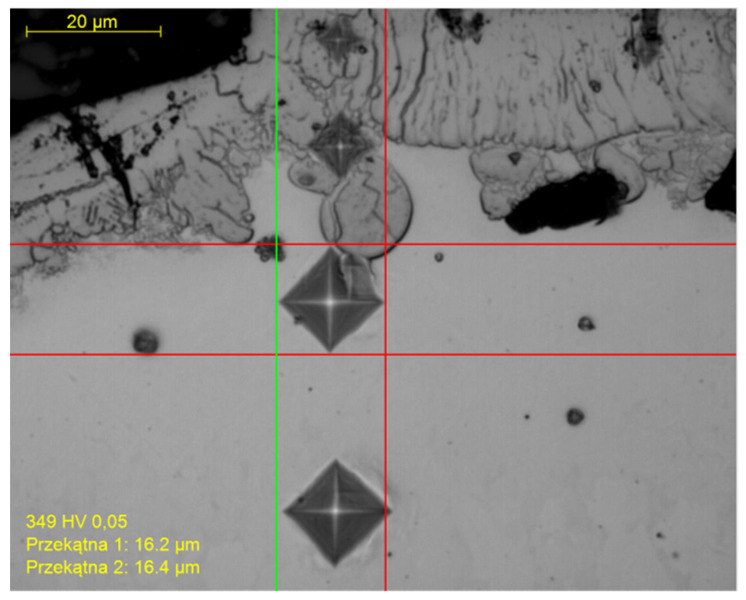
Vickers microhardness Test.

Figure 14 depicts the difference in micro-hardness between the WL, HAZ, and bulk material. Series of Vickers micro-hardness values were conducted under the 0.05 kg load in order to determine the difference in the hardness quantitatively. The Vickers hardness test employs a square-based pyramid diamond indenter with a vertex angle of 136° between the opposing sides, which is driven into the surface of the test item with a predefined force, F. The initial application of the force takes 2 to 8 s, and the test force is held for 10 to 15 s. The indentation’s diagonal lengths are measured following the force’s removal, and the arithmetic means, d, is determined [41]. The results show that the average hardness of the WL is 1786 HV_0.05_, the HAZ is 838 HV_0.05_, and the bulk material after 70 μm deep is 350 HV_0.05_ [17,18]. This increased hardness in the WL and in the HAZ could be associated with metallurgical transformations (such as austenitic-martensitic transformation) during rapid heating and cooling. Another possible explanation could be the presence of oxides, such as rutile-TiO_2_ and V_2_O_5_ [43].

## 4. Conclusions

The current study presented a detailed experimental investigation into EDM’s Ti-6Al-4V ELI processing using a graphite electrode. A full-scale experiment was carried out, with the pulse-on current and time control parameters varying from 9 to 25 A and from 25 to 200 s, respectively. The Material Removal Rate (MRR), Tool Material Removal Rate (TMRR), and Tool Wear Ratio (TWR) were used to estimate machining efficiency and feasibility, while SRa and SRz were used to evaluate surface roughness. Furthermore, the machined surfaces were examined using optical and SEM microscopy to study the surface characteristics and cross-sections, allowing the AWLT thickness to be measured and its chemical composition analyzed. An ANOVA was performed on the indices mentioned above to fully comprehend how process parameters influence the machining result. Finally, the RSM method proposed semi-empirical relationships for the MRR and TMRR, correlating them with the pulse-on current and time. Additionally, by using a microhardness technique, the hardness of the WL was defined. The following are the main findings of the current study:

(1) When it comes to MRR, pulse-on time and the pulse-on current are both factors to consider, but the pulse-on current has the more significant influence. The RSM model, in which I_P_’s contribution is significantly higher than T_on_’s, supports this conclusion as well as the ANOVA analysis. More precisely, the mean MRR increased by 354 percent as I_P_ increased from 9 to 25 A.
(2) The pulse-on current is the most crucial factor in determining the TMRR, while the pulse-on time has a minor and ambiguous role. Furthermore, in the RSM model, the I_P_ term has a magnitude greater than T_on_.
(3) The pulse-on time increases the mean SRa, while the pulse-on current has a more nebulous and ambiguous effect. There is no clear trend or pattern deduced for SRz, so any correlation with I_P_ and T_on_ would be risky at those machining conditions.
(4) Machining conditions have a significant impact on the WL characteristics. With the increasing power and pulse energy, the thickness and homogeneity of the WL are affected. At the same time, the surface quality is poorer in higher machining conditions.
(5) By using SEM microscopy, the EDMed surfaces were depicted in their typical formations. Craters, re-solidified material that forms islets and islets of debris and carbides, pockmarks, as well as areas with developed micro-porosity, were found to be among the cracks. These surface characteristics are affected by the conditions under which they are fabricated.
(6) EDX maps revealed that the aluminum elements evaporate from the machining surface, and the Vanadium takes its place by increasing the machining conditions. That may be an indication that the subsurface contains beta phase titanium.
(7) Additionally, there are indications of material diffusion from the electrode to the materials’ WL.
(8) The average microhardness of the WL is 1786 HV_0.05_, HAZ is 838 HV_0.05_ while the microhardness of the bulk material after 70 μm deep is stable and about 350 HV_0.05._

## Figures and Tables

**Table 1:** Mechanical properties of the Ti-6Al-4V ELI, and the graphite electrode.

Material	Ti Grade 23	Graphite Electrode
Density [g/cm^3^]	4.43	1.77
Melting Point [°C]	1604–1600	3300
Electrical Resistively [μΩcm^−1^]	53.3	500
Hardness [HB]	326	10
Thermal Conductivity [W/mK]	16.70	168

**Table 2:** Chemical composition of Ti-6Al-4V ELI.

Ti	C max (%)	Fe max (%)	H max (%)	N max (%)	O max (%)	V (%)	Al (%)
Bal.	0.08	0.25	0.0125	0.03	0.13	3.5–4.5	5.5–6.5

**Table 3:** Machining Conditions.

Machining Conditions	Level 1	Level 2	Level 3	Level 4
Pulse-on current I_p_ [A]	9	13	17	25
Pulse on-time T_on_ [μs]	25	50	100	200
Close Circuit Voltage V_c_ [V]	30			
Duty Factor	0.5			
Dielectric	Synthetic hydrocarbon fluid			
Dielectric Flushing	Side flushing with pressure			
Dielectric Flushing Pressure [MPa]	0.7 (Constant under the whole conditions)			

**Table 4:** Experimental result.

I_P_ [A]	T_on_ [μs]	MRR [mm^3^/min]	TMRR [mm^3^/min]	TWR [%]	SRa [μm]	SRz [μm]	AWLT [μm]
9	25	0.36	0.60	0.66	10.81	96.31	9.57
9	50	0.27	0.71	1.07	13.18	125.58	11.18
9	100	0.16	0.35	0.86	12.14	117.46	13.98
9	200	0.25	0.27	0.42	15.01	128.80	25.42
13	25	0.54	0.94	0.70	12.99	117.07	10.32
13	50	0.62	1.30	0.84	14.64	125.76	13.83
13	100	0.56	1.11	0.79	17.75	159.84	13.75
13	200	0.43	0.77	0.72	14.79	135.59	26.16
17	25	0.85	1.31	0.62	12.01	112.08	8.63
17	50	0.70	1.52	0.87	14.76	135.38	13.09
17	100	0.84	1.42	0.68	15.13	139.86	21.76
17	200	0.58	0.97	0.67	16.84	136.15	22.99
25	25	1.08	2.18	0.80	13.92	120.59	35.79
25	50	0.98	2.29	0.93	16.03	158.36	24.05
25	100	1.45	2.35	0.65	17.55	134.63	20.57
25	200	1.23	2.03	0.66	15.24	138.24	27.80

## Data Availability

The data presented in this study are available on request from the corresponding author.
